# Ovine fetal testis stage-specific sensitivity to environmental chemical mixtures

**DOI:** 10.1530/REP-21-0235

**Published:** 2022-01-11

**Authors:** Richard G Lea, Beatrice Mandon-Pépin, Benoit Loup, Elodie Poumerol, Luc Jouneau, Biola F Egbowon, Adelle Bowden, Corinne Cotinot, Laura Purdie, Zulin Zhang, Paul A Fowler, Kevin D Sinclair

**Affiliations:** 1 University of Nottingham, Sutton Bonington Campus, Loughborough, UK; 2 Université Paris-Saclay, UVSQ, INRAE, BREED, Jouy-en-Josas, France; 3 James Hutton Institute, Craigebuckler, Aberdeen, UK; 4 University of Aberdeen, Institute of Medical Sciences, Aberdeen, UK

## Abstract

Exposure of the fetal testis to numerous individual environmental chemicals (ECs) is frequently associated with dysregulated development, leading to impaired adult reproductive competence. However, ‘real-life’ exposure involves complex mixtures of ECs. Here we test the consequences, for the male fetus, of exposing pregnant ewes to EC mixtures derived from pastures treated with biosolids fertiliser (processed human sewage). Fetal testes from continuously exposed ewes were either unaffected at day 80 or exhibited a reduced area of testis immunostained for CYP17A1 protein at day 140. Fetal testes from day 140 pregnant ewes that were exposed transiently for 80-day periods during early (0–80 days), mid (30–110 days), or late (60–140 days) pregnancy had fewer Sertoli cells and reduced testicular area stained for CYP17A1. Male fetuses from ewes exposed during late pregnancy also exhibited reduced fetal body, adrenal and testis mass, anogenital distance, and lowered testosterone; collectively indicative of an anti-androgenic effect. Exposure limited to early gestation induced more testis transcriptome changes than observed for continuously exposed day 140 fetuses. These data suggest that a short period of EC exposure does not allow sufficient time for the testis to adapt. Consequently, testicular transcriptomic changes induced during the first 80 days of gestation may equate with phenotypic effects observed at day 140. In contrast, relatively fewer changes in the testis transcriptome in fetuses exposed continuously to ECs throughout gestation are associated with less severe consequences. Unless corrected by or during puberty, these differential effects would predictably have adverse outcomes for adult testicular function and fertility.

## Introduction

Exposure to environmental pollutants during fetal, neonatal, and/or adult life is associated with adverse effects on male reproductive development and function (Sumner *et al.* 2020). Chemical release into the environment has increased markedly since the 1950s (>140,000 chemicals) and this is associated with adverse temporal trends in human male reproductive health (Landrigan 2017). Meta-analyses now confirm that human sperm counts have declined by 50% over 70 years (Carlsen *et al.* 1992, Swan *et al.* 2000, Levine *et al.* 2017). Concomitant with these temporal changes, incidences of testicular cancer in young adults and malformations in male newborns (cryptorchidism and hypospadias) have also increased (Skakkebaek *et al.* 2016, Park *et al.* 2018). In support of an environmental cause, the occupational exposures of mothers to chemical pollutants have been associated with reduced semen quality in their offspring (Istvan *et al.* 2021). Exposure studies in sheep, dogs, and rodents are similarly indicative of pollutant effects on male reproductive health (Paul *et al.* 2005, Salian *et al.* 2011, Sumner *et al.* 2020). In addition, animal model studies indicate that male reproductive disorders are linked and originate following exposure during fetal life (Skakkebaek *et al.* 2001, Wohlfahrt-Veje *et al.* 2009). The majority of studies have focussed on single chemicals and, in some cases, at doses much higher than environmental exposure levels. Since environmental pollutants constitute a complex mixture, extrapolating from single chemical effects to real-life exposure is problematic.

To investigate real-world exposures, pregnant ewes are grazed on pastures treated with biosolids fertiliser generated from processed human sewage sludge. This is recognised agricultural practice worldwide and the fertiliser contains chemical pollutants which, in combination, represent exposure in humans. A wide range of chemical types have been detected in biosolids and in maternal and fetal livers collected from ewes that had grazed-treated pastures (Lea *et al.* 2016, Viguie *et al.* 2020). Although directly equating changes in liver chemical load with concentrations in the soil are problematic, exposure has been linked to an increased chemical burden in maternal livers and developmental effects in the mid-gestation male fetal testis. These include reduced numbers of gonocytes, Sertoli and Leydig cells, and a parallel reduction in fetal inhibin A and testosterone (Paul *et al.* 2005). Extending gestational exposure to day 1 neonates and to 7 months in offspring reduced germ cell numbers and induced Sertoli cell-only tubules in cohorts of neonatal and adult animals. In adults, reduced Sertoli cell numbers were also reported (Bellingham *et al.* 2012, Elcombe *et al.* 2021). Biosolids exposure is also associated with adverse effects on the fetal ovary, thyroid, and hypothalamo–pituitary axis as well as in the adult liver (Fowler *et al.* 2008, Bellingham *et al.* 2009, 2010, Hombach-Klonisch *et al.* 2013, Lea *et al.* 2016, Filis *et al.* 2019). Taken together, a conclusion that exposure to chemicals not only impacts the developing fetus but also has longer-term consequences for adult wellbeing is inescapable.

**Figure 1:**
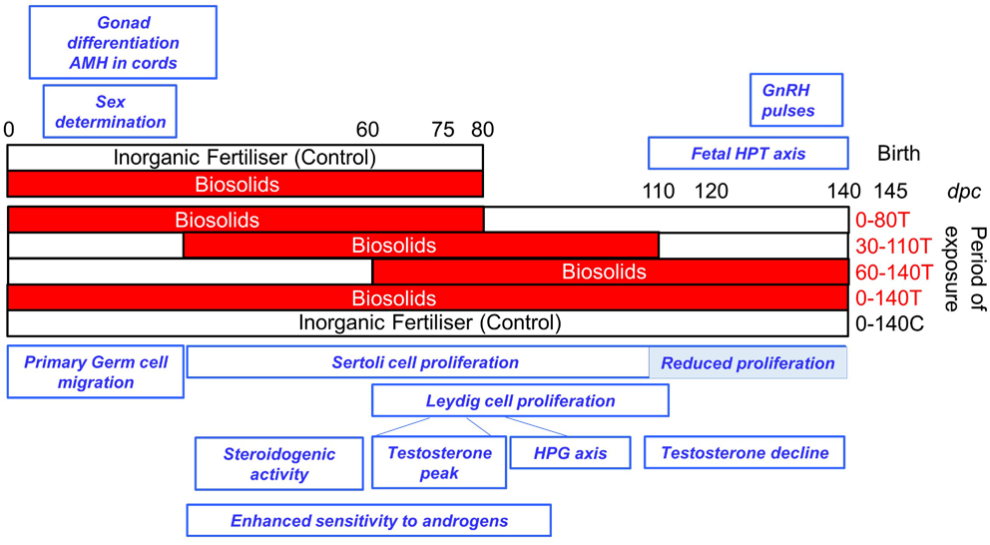
Experimental design to investigate the effects of biosolids exposure on the fetal testis. Pregnant ewes were sacrificed at day 80 (2 cohorts) or day 140 (5 cohorts) of gestation. In the day 80 group, ewes were exposed to biosolids-treated pastures or pastures fertilised with inorganic fertiliser. In pregnant ewes maintained to day 140, exposure to biosolids-treated pastures occurred from: days 0 to 140 (0-140T, continuous exposure), days 0 to 80 (0-80T, early exposure), days 30 to 110 (30-110T, mid-exposure), and days 60 to 140 (60–140T, late exposure). Control group pregnant ewes (C) were maintained on pastures treated with inorganic fertiliser from days 0 to 140 of gestation (0–140C). For array transcriptomics, 0–80T, 0–140T, and 0–140C groups were used. The main events of differentiation in the fetal testis are described in the blue rectangles. Key references: Sertoli cell proliferation: (Hochereau-de Reviers *et al.* 1995), steroidogenic activity/testosterone: (Pomerantz & Nalbandov 1975, Quirke *et al.* 2001), fetal HPT function: (Mesiano *et al.* 1991, Roselli *et al.* 2016).

We previously reported that exposing ewes for overlapping 80-day periods that encompass early (0–80 days), mid (30–110 days), and late (60–140 days) gestation induce differential and adverse changes in the fetal ovary (Lea *et al.* 2016). Since these 80-day periods also encompass critical developmental stages in the developing fetal testis (Fig. 1), the current study was designed to determine the effects of transient and consistent exposures on the developing male. Given the continuum of testis development, days 0, 30, and 60 fetal testes will have different cellular compositions largely reflecting periods of Sertoli and Leydig cell proliferation. Since this depends on gestational age, we postulated that the 80-day exposure periods will differentially impact developmental processes including Sertoli and Leydig cell proliferation. Our primary morphological studies therefore focussed on these cell types. Here, we report that exposure during the first third of gestation induced transcriptomic changes measurable in the late gestation fetal testis and exposure in the last third of gestation induced broader phenotypic changes in the male fetus.

## Methods

### Animals, treatment groups, and tissue collection

All animal experimental protocols were approved by the James Hutton Institute’s Local Ethical Committee and licensed by the United Kingdom’s Animals Scientific Procedures Act 1986 (Project license: 60/3356). All procedures were performed in accordance with relevant guidelines and regulations.

The treatment of pastures with biosolids and grazing of sheep were carried out as previously described (Lea *et al.* 2016). In brief, pastures were fertilised with a single treatment of thermally dried sewage sludge (2.25 metric tons of dry matter/ha; treated: T) or with inorganic fertiliser balanced for nitrogen (225 kg/ha/year; control: C). Experimental animals were maintained on either C or T pastures. Mature Texel ewes were allocated to one of seven treatment groups (Fig. 1) randomly keeping body condition score as consistent as possible. Although initial group size comprised approximately 14 pregnant ewes, the results reported concern only those carrying at least 1 male fetus.

Two groups were either exposed to biosolid treated or control pastures from mating to sacrification at mid-gestation (day 80). The remaining five groups were all maintained to sacrification at late gestation (day 140) and comprised one group grazed on C pastures throughout (0–140C), one group exposed to T pastures throughout (0–140T), and three transiently exposed groups where exposure was limited to 80-day periods during early (0–80 days), mid (30–110 days), or late (60–140 days) gestation (Fig. 1). Pregnancies were established by mating ewes with Texel rams at the second synchronized estrus following the withdrawal of progestagen sponges (chronolone, 30 mg; Intervet, Cambridge, UK). Pregnant ewes were either maintained on T pastures or transferred between C and T pastures (and back again), as appropriate. All ewes were habituated to being moved between pastures, thus minimising stress. When pregnant ewes were moved, they were maintained on a separate pasture for a few days to minimise C pasture contamination by faeces and urine.

All pregnant ewes were euthanised according to Schedule 1 protocols (UK Animals Scientific Procedures Act, 1986). Samples and data (organ weights, etc.) were collected and processed according to our standard protocols (Bellingham *et al.* 2013, Lea *et al.* 2016). In brief, fetal anogenital distance (AGD) and selected organ weights were measured and recorded. One testis per fetus was analysed for histology and the second for transcriptomic studies. Testes were Bouin’s fixed for histological analysis or snap-frozen in liquid nitrogen and stored at −80°C for RNA extraction. Maternal and fetal liver sample processing and chemical measurements were reported by (Lea *et al.* 2016).

### Testis immunohistochemistry and histomorphology

Bouin’s-fixed testes were processed for immunohistochemistry: Sertoli cells (anti-Mullerian hormone; AMH), Leydig cells (steroidogenic enzymes: CYP11A1, CYP17A1), and proliferation (Ki67). Established immunohistochemistry protocols were applied (Paul *et al.* 2005, Andrade *et al.* 2013) including epitope retrieval (microwaving: 3 × 5 min in 0.01 M citrate buffer pH 6.0) and application of the Vectastain ABC universal Elite kit (2B Scientific Ltd, Stonesfield, UK) protocol. Tissue sections were incubated with primary antibodies for 1 h at room temperature: (a) polyclonal goat AMH (Santa Cruz: 0.125 µg/mL); (b) monoclonal mouse anti-human Ki67 (Clone MIB-1: Dakocytomation: 0.8 µg/mL); (c) polyclonal rabbit anti-CYP11A1 (Merck Millipore: 4 µg/mL); (d) polyclonal rabbit anti-CYP17A1 (gift: Professor Ian Mason, Edinburgh: 10 µg/mL). Negative controls comprised incubation with non-specific mouse, rabbit, or goat IgG. Sections were visualised by incubation with DAB chromagen according to kit instructions (2B Scientific Ltd). The area of cells positively stained for CYP17A1 or CYP11A1 was measured as a percentage of total interstitial area (Image-Pro Plus: Media Cybernetics, Maryland, USA). Twenty randomly selected digital images (×400) were analysed by a single observer (BE) while blinded to the group being examined.

Sertoli cell numbers were determined stereologically (Paul *et al.* 2005, Andrade *et al.* 2013). In brief, 5-µm sections were subjected to immunohistochemistry for AMH. Forty images at ×630 magnification were captured from one testis cross-section using Leica software, ten per pole. Images were overlaid with a 432 point grid (Image-Pro Plus: Media Cybernetics). Sertoli cells across an intersection were counted and this total point value was expressed as a percentage of the maximum count across all 40 images (40 × 432 = 17,280). Testis weight occupied by Sertoli cells was calculated (e.g. 1 g testis with an average point count of 10% = 0.1 g of Sertoli cells). A weight-to-volume conversion was applied (1 g = 1 cm^3^) to generate the absolute volume (AV) of the testis occupied by Sertoli cells. The average Sertoli cell diameter was measured (Image-pro plus) and the mean nuclear volume (MNV) of each Sertoli cell was determined from the formula (4/3π)r^3^. The total number of Sertoli cells per testis was calculated (AV (μm^3^)/MNV(μm^3^)) and adjusted to account for testis weight.

### Measurement of testosterone

Fetal serum concentrations of total testosterone (bound and unbound) were measured using the automated ADVIA Centaur XP competitive immunoassay system (Siemens Healthcare Diagnostics), as previously reported (Lea *et al.* 2016). Total serum testosterone assay sensitivity was 0.35 nmol/L and the mean intra- and inter-assay CV values were 4.4 and 6.2%, respectively.

### Fetal testicular RNA extraction

RNA, DNA, and protein were extracted from fetal testis using an AllPrep DNA/RNA/Protein mini kit (Qiagen Ltd.). Samples were homogenised and processed as previously described (Lea *et al.* 2016). The manufacturer’s instructions were followed with the optional on-column DNase digestion included. Samples were homogenised in 600 μL RNeasy lysis buffer for tissues (RLT) buffer for 2 min at 30 Hz, centrifuged after which the lysate was added to an ALLprep DNA column. After centrifugation, the column flow-through was processed by passing through an RNeasy spin column according to kit instructions. In brief, RNA wash buffer (RW1) buffer was centrifuged through the column after which a DNase I incubation mix was added followed by a 15-min incubation at room temperature. The column was then washed/centrifuged with RW1 buffer (350 μL) and 2 × RNA purification and elution buffer (RPE) buffer (500 μL) after which the addition of 30 μL of RNase-free water followed by centrifugation yielded RNA containing flow through for cDNA synthesis.

### Customised ovine microarray and pathway analyses

Transcriptome analysis was conducted using a custom 15K Agilent oligo sheep microarray generated as previously described (Lea *et al.* 2016). In brief, a catalogue Sheep Gene Expression Microarray 8 × 15K (G4813A-019921) was modified by adding new target sequences and removing redundancies. The latter was identified from sheep ESTs assembly and oligo annotations performed by Sigenae (http://www.sigenae.org/). Several different oligonucleotides were used to target sheep contigs, and the best annotated of these at the 3’ end were conserved (2 per contig max). Other oligos specific for the same transcript were removed from the array. Agilent ‘GE Probe Design’ eArray workflow and tools (https://earray.chem.agilent.com/earray/) were used for oligo design. All 1500 new oligos, and those remaining on the original Agilent array, were annotated using Sigenae SigReannot tool49. The array was enriched with genes initially identified in the fetal sheep gonad and completed with control genes known to be expressed in developing fetal gonads from both sexes. The final version of the array comprised 7500 different genes and labelling and hybridisation were performed at the ‘Plate-forme Biopuces et Sequencage’ (http://www-microarrays.u-strasbg.fr/). Following one-colour labelling and hybridisation using the Quick Amp Labelling kit (Agilent, 5190–0442) and One- colour RNA Spike-in Kit (Agilent, 5188–5282), arrays were scanned with the Agilent DNA Microarray Scanner Model G2565B. Image analysis was performed with Agilent Feature Extraction software v9.5.3.1.

### Gene ontology enrichment

Identified sheep differentially expressed probes were analysed with Gene Ontology (GO) and Kyoto Encyclopedia of Genes and Genomes (KEGG) pathway membership with Database was performed using the DAVID Bioinformatic Database 6.8 (https://david.ncifcrf.gov/). These analyses and pathways were considered significant for a Benjamini corrected enrichment score of less than 0.05. Further analysis of gene pathways utilised the Ingenuity Pathway Analysis software (http://www.ingenu ity.com/). This pathway analysis system uses the same hypergeometric test as DAVID, and topology-based output was used to assess the biological processes impacted by biosolids exposure (Nguyen *et al.* 2019). We used the eXploring Genomic Relations web tool (http://galahad.well.ox.ac.uk:3040/) under default parameters and with lists of differentially expressed genes tested for enrichment of annotations (Fang *et al.* 2016).

### Pathway and functional analysis of differentially expressed genes (DEGs)

To further understand biological functions and pathways, sheep differentially expressed transcripts (false discovery rate (FDR) ≤ 5%; LogFC threshold ± 0.2) were functionally annotated based on GO terms and KEGG pathway through the DAVID ontology database. Uncharacterized, putative genes and redundant probes were removed so that 1596 (0–80T) and 1567 (0–140T) official gene name symbols were subjected to DAVID analyses. To increase the depth of genes with GO annotations, *Homo Sapiens* genome annotation was used as background and statistically enriched biological processes and molecular functions were obtained in which the proteins are involved (Ha *et al.* 2015). The transcripts were classified as: biological process, cellular component, and molecular function. An individual transcript may be represented in several categories.

### Validation of transcriptomic data by qPCR

Using the same samples used for microarray, transcript expression was confirmed by real-time PCR (Lea *et al.* 2016). DNase-treated RNA was used to synthesise cDNA (Transcriptor First Strand cDNA Synthesis Kit: Roche). Standard curves were generated from pooled cDNA (1:5 serial dilutions), and all cDNA samples were diluted at 1:50 in dH_2_O. Primers and probes were designed using Primer3 software, and DNA probes were synthesised with a 5′-FAM fluorophore and a 3′-TAMRA quencher (Eurofins, Ebersberg, Germany). Genbank accession numbers and probe and primer sequences are listed in Supplementary Table 1 (see section on supplementary materials given at the end of this article). Samples were run in triplicate using the LightCycler® 480 Probes Master (Roche), and ‘no template controls’ were included as standard. Reaction mixtures and cycling conditions were as previously reported (Lea *et al.* 2016). All qPCR data were analysed using Roche LightCycler480 software and normalised using the geNorm method. Three housekeeping genes (*GAPDH, HPRT, YWHAZ*) were tested for stability using geNorm, Normfinder, and ANOVA analysis.

### Statistical analysis

Morphometric data comprising fetal mass, organ weights (testis, thyroid, adrenal, liver), and AGD were analysed using Generalized Linear Regression (GLR: Genstat statistical package version 20; https://www.vsni.co.uk/). Since males were from singleton and twin pregnancies, data were adjusted for litter size (fixed effects: number of fetuses and treatment, random effect: ewe). The Bonferroni multiple comparisons adjustment was applied, and between-group differences were identified. Sertoli cell numbers and Leydig cell staining were also analysed by GLR. Sertoli and Leydig cell data are presented as scatter plots with means (GraphPad Prism Prism, version 8). Fold changes were presented as positive or negative values relative to controls.

For gene array, data processing and analysis were conducted using Biocondutor packages suite (http://www.bioconductor.org/index.html) and LIMMA package50 with the R statistical programme. Raw median signal from Feature Extraction array files was used as non-processed signal and log_2-_transformed. Background was then subtracted locally, and intra-array normalisation was performed by subtracting the array median signal from each spot signal on the same array. Multiple testing corrections were applied, and differentially expressed transcripts were considered under a FDR of 5% (Benjamini & Hochberg 1995).

## Results

### Effects of biosolids exposure on the fetal urogenital tract and testosterone

At day 80, male fetal mass was not altered by continuous exposure to biosolids and there were no treatment effects on AGD or organ weights (i.e. testis, thyroid, adrenal, and liver: Table 1). In contrast, day 140 male fetuses from ewes exposed from 60 to 140 days of gestation (60–140T group) had a lower fetal mass than non-exposed controls (0–140C), continuously exposed fetuses (0–140T) and fetuses exposed mid-gestation (30–110T) (*P*< 0.001). AGD was shorter in the 60–140T group than controls (*P*< 0.05) (Table 1). The late exposure group (60–140T) also had smaller adrenals (*P*< 0.05), smaller testes (*P*< 0.001), and lower levels of testosterone (*P*< 0.01) compared to control non-exposed fetuses (Table 1).

**Table 1:** Morphology (days 80 and 140) and endocrinology (day 140) of male fetuses following exposure to biosolids-treated pastures. Values represent predicted means ± S.E.M. Experimental groups: C = control, T = exposed. Paired organs were combined into a single weight. Data were fitted to a general linear model, adjusting for litter size, and analysed by general linear regression (Genstat). Between-group differences were analysed by Bonferroni (day 140).

	Exposure groups								
	Day 80 fetuses			Day 140 fetuses					
	0–80C	0–80T	*P*	0–140C	0–140T	0–80T	30–110T	60–140T	*P*
Morphology									
No. ewes > 1 male fetus (D80^A^, D140^B^)	8	9		11	9	7	8	11	
No. fetuses^C^	13	11		15	12	9	12	15	
Fetal mass (g, D80; kg D140)	346.8 ± 11.66	318.2 ± 12.69	NS	5.39 ± 0.22^a^	5.24 ± 0.26^a^	5.01 ± 0.31^ab^	5.32 ± 0.24^a^	**4.05 ± 0.24^b^**	<0.001
AGD (mm)	47.07 ± 0.66	47.77 ± 0.71	NS	151.6 ± 3.38^a^	144.6 ± 4.06^ab^	150.7 ± 4.67^ab^	142.4 ± 3.61^ab^	**134.9 ± 3.56^b^**	<0.05
Testis (mg)	155.7 ± 9.4	141.4 ± 10.2	NS	2590 ± 112.3^a^	2534 ± 128.3^a^	2521 ± 155.5^a^	2192 ± 120.3^a^	**1677 ± 118.3 ^b^**	<0.001
Thyroid (mg)	141.5 ± 12.84	128.7 ± 14.0	NS	1370 ± 107.2	1354 ± 128.8	1117 ± 148.0	1249 ±119.8	1328 ± 113.0	NS
Adrenals (mg)	126.6 ± 8.5	132.4 ± 9.2	NS	647.8 ± 28.47^a^	572.9 ± 32.53^ab^	549.0. ± 39.44^ab^	591.5 ± 30.50^ab^	**505.1 ± 29.99^b^**	<0.05
Liver (g)	21.21 ± 0.6	19.6 ± 0.7	NS	132.0 ±7.97	130.9 ± 9.11	134.7 ± 11.04	149.8 ± 8.54	117.3 ± 8.40	NS
Fetal endocrinology									
Fetal testosterone				3.60 ± 0.21^b^	3.75 ± 0.28^b^	3.45 ± 0.30^ab^	3.52 ± 0.23^b^	**2.54 ± 0.23 ^a^**	<0.01

^A^Ewes at day 80: 82% (14/17) carried twin pregnancies and the remaining pregnancies were triplets (*n* = 3); ^B^Ewes at day 140: 69% (32/46) carried twin pregnancies and remaining pregnancies were singles (*n* = 7) and triplets (*n* = 7); ^C^Fetuses were used from all pregnant ewes. Differing superscripts indicate differences between groups and values in bold indicate differences relative to 0–80C or 0–140C.

AGD, anogenital distance.

### Effects of biosolids exposure on testis development

**Figure 2:**
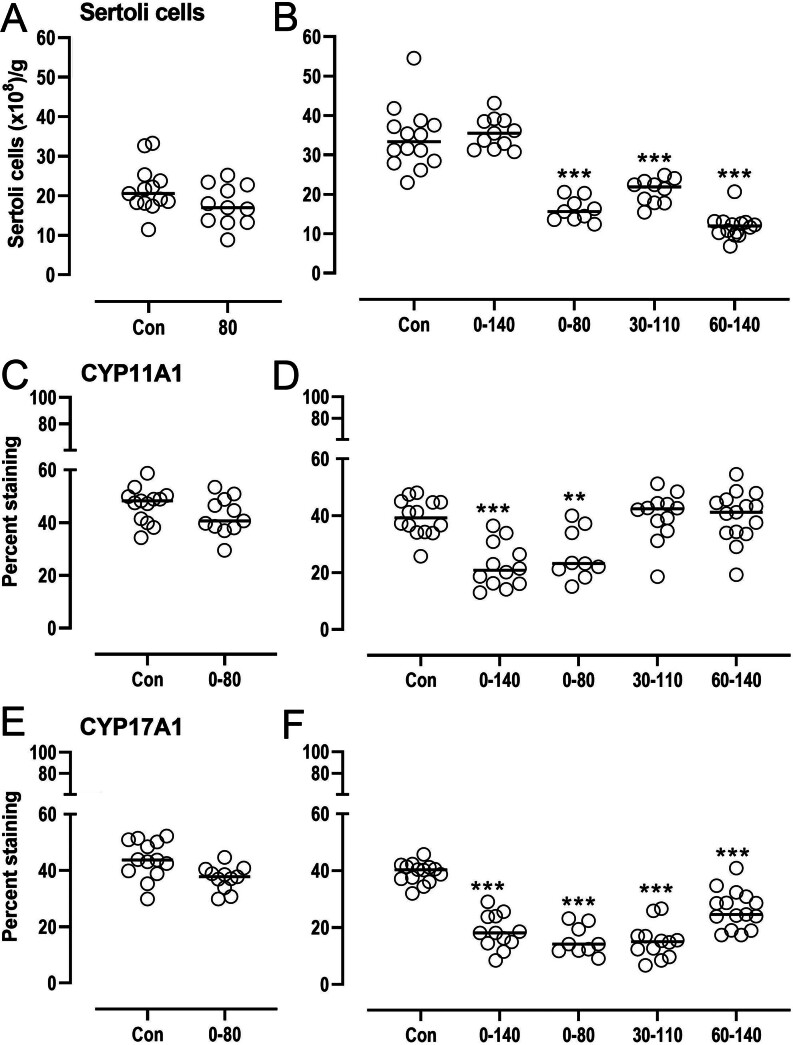
Disturbance in Sertoli cells numbers and area positive for steroidogenic cells in fetal testes following continuous or transient exposure of pregnant ewes to biosolids. Fetal testes were collected at days 80 or day 140 of gestation. Day 80 testes were derived from control (con) non-exposed and continuously exposed mothers. Day 140 testes were derived from control (con) non-exposed mothers, from mothers exposed continuously (0–140) and from mothers exposed for a period of 80 days limited to early (0–80 days), mid (30–110 days), and late gestation (60–140 days). Sertoli cells (A: day 80, B: day 140) were identified by AMH staining, quantified stereologically, and expressed as numbers per gram of testis. Steroidogenic interstitial cells were identified by immunohistochemistry quantified by image analysis as per cent nucleated interstitial area stained for CYP11A1 (C: day 80, D: day 140) and CYP17A1 (E: day 80, F: day 140). Each symbol corresponds to an individual control or exposed animal. The horizontal line indicates the mean for each group. Day 140 data were fitted to a generalized linear regression model, adjusting for litter size (fixed effects: number of fetuses and treatment, random effect: ewe) (Genstat). Treatment effect: *P*< 0.001. Between-group differences were analysed by Bonferroni: asterisks indicate significant differences with control non-exposed group.

*Day 80:*continuous maternal exposure to biosolids had no effect on AMH-positive Sertoli cell numbers per gram of testis (Fig. 2A and Supplementary Fig. 1A). There was no treatment effect on per cent nucleated area stained for the steroidogenic enzyme CYP11A1 (Fig. 2C and Supplementary Fig. 1C) or CYP17A1 (Fig. 2E and Supplementary Fig. 1D).

*Day 140:*Sertoli cell numbers per gram of testis were reduced by transient exposure of the mother to biosolids, regardless of when exposure occurred. In contrast, continuous exposure from 0 to 140 days had no effect on Sertoli cell numbers (Fig. 2B) (*P*< 0.001). Positive staining for Ki67 was indicative of Sertoli cell proliferation as expected at this developmental stage (Supplementary Fig. 1B). The per cent testicular area stained for CYP11A1 was reduced in the 0–140T (*P*< 0.001) and 0–80T (*P*< 0.01) exposure groups (Fig. 2D), whereas staining for CYP17A1 was reduced in continuous and all transient exposure groups (Fig. 2F).

### Effects of biosolids exposure on the testis transcriptome

**Figure 3:**
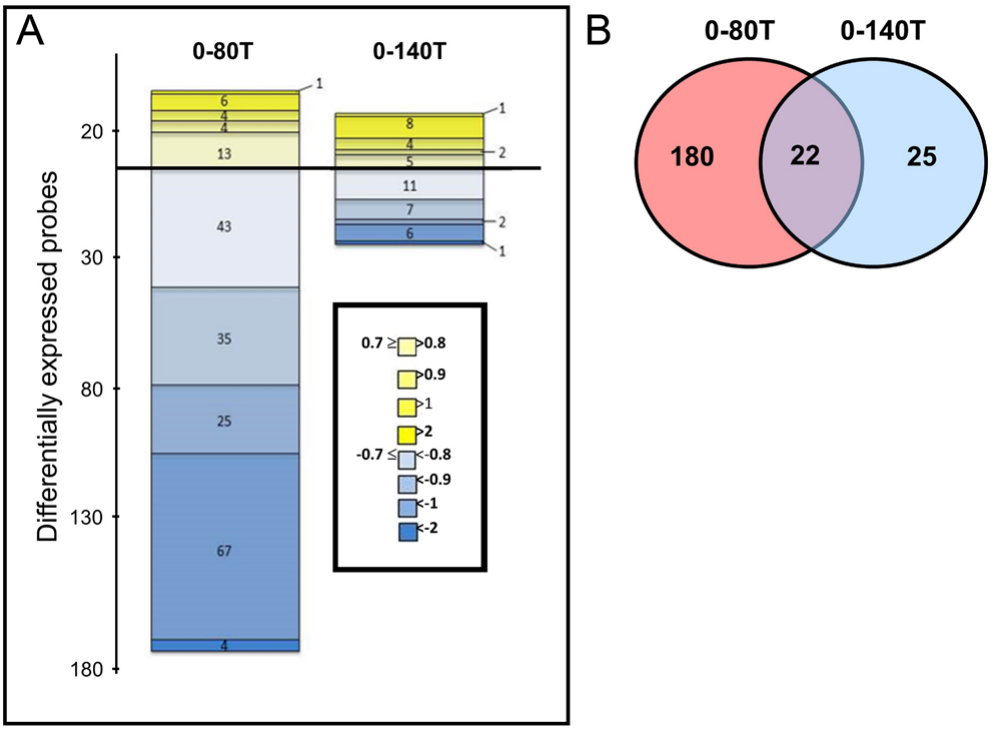
Analysis of differentially expressed (relative to control) fetal testis genes following maternal exposure to biosolids. At day 140, more genes were altered relative to control following biosolids exposure in the 0–80T than in the 0–140T exposure group. (A) Numbers and fold change of differentially expressed genes. Only probes that met an FDR of 5% and a threshold of ±0.7 on the log_2-_transformed fold change (LogFC) are displayed. Probes are represented according to their positive (yellow) or negative (blue) fold change. (B) Venn diagram showing the number of differentially expressed transcripts unique to and common to both exposure groups. Day 140 testes from 0 to 80T exposed fetuses had >8× the number of differentially expressed transcripts compared to testes from the 0 to 140T group.

Gene array analysis was carried out on three groups of fetuses at 140 days: 0–140C, 0–140T, and 0–80T. Analyses revealed that 249 transcripts (3.3% of 7500) were differentially expressed (*P* < 0.05 after Benjamini–Hochberg multiple testing correction (FDR at 5%) and absolute fold change > 1.5) between controls and exposed fetal testes (Fig. 3A). Of these transcripts, 47 were differentially expressed in testes from continuously exposed mothers (0–140T). In contrast, 202 transcripts were differentially expressed in the 0–80T group (Fig. 3A). A total of 22 differentially expressed genes were common to both the 0–80 and 0–140 exposure groups (Fig. 3B). In the exposure groups, the majority of genes were downregulated, particularly in the 0–80T group (0–80T, 174/202: 86% vs 0–140T, 27/47, 57%) (Fig. 3A and Supplementary Table 2). The complete raw data transformed by the platform (processed signal) are provided as Supplementary Table 3.

**Figure 4:**
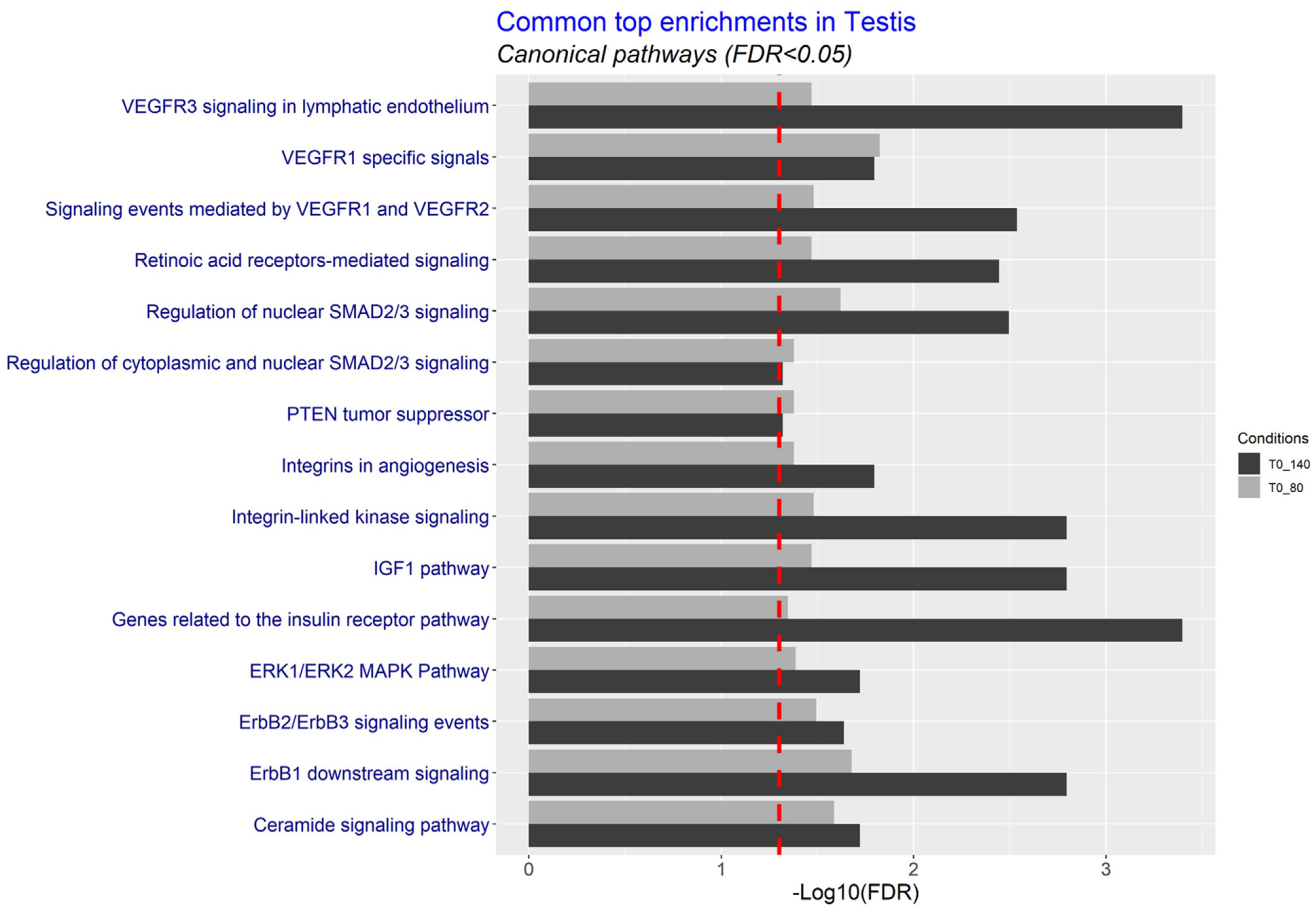
Common canonical testicular pathways following maternal exposure to biosolids in 0–80T and 0–140T groups. Common enrichment probes coding for canonical pathways were analysed using the eXploring Genomic Relations (XGR) web tool.

**Figure 5:**
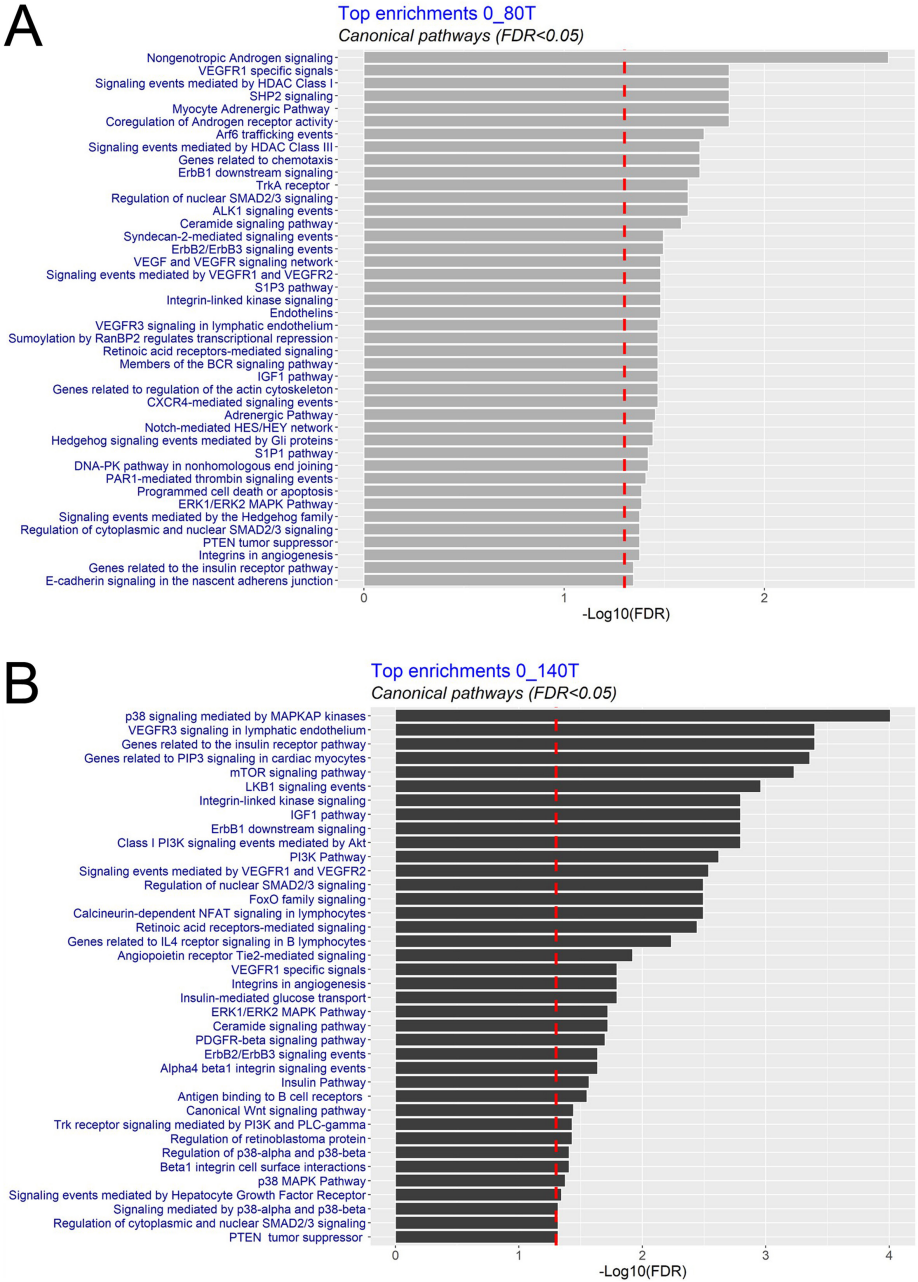
Functional analysis of differentially expressed testis transcripts following maternal exposure to biosolids. Top canonical pathways are shown in (A) 0–80T and (B) 0–140T exposure groups. Analysis was carried out using the eXploring Genomic Relations (XGR) web tool.

The functional analysis of differentially expressed transcripts common to both exposure groups highlighted differentially expressed genes associated with VEGF and signalling pathways: SMAD2/3, IGF1, ERK1/ERK2 MAPK, ErbB1,2,3 (Fig. 4). Differentially expressed transcripts specific to the 0–80T exposure group were associated with a range of cell specific functions: androgen signalling, angiogenesis, and cell signalling (Fig. 5A). In the lesser perturbed group (0–140T), differentially expressed transcripts were associated with cell signalling, metabolism (e.g. insulin receptor pathway), and angiogenesis (Fig. 5B).

Topological analyses by ingenuity pathway analysis revealed differentially expressed gene networks associated with drug metabolism, disorders of haematological neurological, cardiovascular systems, and metabolic disease (Table 2). High scoring gene networks in the 0–140T group were primarily linked to cell function and these networks were also identified in the 0–80T group (Table 2). Biological functions primarily highlighted in the 0–80T group were linked with cancer and reproductive system disease, dermatological disease, and genetic disorders (Table 3). The latter two functions were also highlighted in the 0–140T group along with inflammatory disease and skeletal/muscular disorders. Molecular, cellular, and physiological functions in the 0–140T group were low scoring. However, in the 0–80T group, organismal and tissue development along with cell morphology, assembly, and organisation were high-scoring networks (Table 3).

**Table 2:** Functional analysis of differentially expressed transcripts representative of specific biological pathways.

Treatment group	Associated network functions^a^	Score
0–80T	1. Cellular movement, cell morphology, drug metabolism 2. Cell death, hematological system development and function, cell-mediated immune response 3. Cell cycle, hair and skin development and function, connective tissue development and function. 4. Developmental disorder, neurological disease, cardiovascular system development and function 5. Genetic disorder, metabolic disease, cellular assembly and organisation	44 42 33 27 25
0–140T	1. Cell signalling, small molecule biochemistry, inflammatory response 2. RNA post-transcriptional modification, cell to cell signalling and interaction, cellular assembly and organisation 3. Embryonic development, organ development, organismal development 4. Endocrine system disorders, gastrointestinal disease, genetic disorder 5. Lipid metabolism, small molecule biochemistry, connective tissue disorders	34 30 3 3 3

^a^Biological pathways identified by ingenuity pathway analysis (IPA) software (FDR < 7%).

**Table 3:** Biological functions associated with testicular differentially expressed genes following biosolids exposure.

Biological functions	Treatment groups			
	0–80T		0–140T	
	*P*	No. mols	*P*	No. mols
Diseases and disorders				
Dermatological disease/conditions	9.47E−04 – 5.96E−03	23	3.83E−05 – 4.58E−02	8
Genetic disorder	9.47E−04 – 4.86E−02	25	9.47E−04 – 5.96E−03	23
Cancer	1.06E−04 – 3.66E−02	45		
Reproductive system disease	3.83E−05 – 4.67E−02	27		
Gastrointestinal disease	9.47E−04 – 4.95E−02	11		
Connective tissue disorders			3.83E−05 – 4.67E−02	3
Inflammatory disease			9.47E−04 – 4.86E−02	13
Skeletal and muscular disorders			9.47E−04 – 4.95E−02	12
Molecular and cell functions				
Cell morphology	1.75E−04 – 3.01E−02	20	1.16E−03 – 4.48E−02	9
Cell assembly and organisation	3.24E−04 – 3.66E−02	29		
Cellular compromise	3.24E−04 – 3.66E−02	9		
Lipid metabolism	5.38E−04 – 3.38E−02	8		
Molecular transport	5.38E−04 – 3.66E−02	12		
Gene expression			4.53E−04 – 4.10E−02	3
Cell function and maintenance			8.66E−04 – 4.29E−02	8
Cell death			1.12E−03 – 4.67E−02	8
Cellular movement			1.41E−03 – 4.48E−02	6
Phyiological system development and function				
Organismal development	2.62E−04 – 3.66E−02	37	1.12E−03 – 3.72E−02	8
Cardiovascular system	1.30E−03 – 2.93E−02	14	1.84E−03 – 3.72E−02	5
Tissue development	8.03E−04 – 3.66E−02	34		
Hair and skin	1.54E−03 – 3.10E−02	10		
Renal and urological	1.81E−03 – 3.66E−02	13		
Reproductive system			3.97E−04 – 2.56E−02	6
Organ development			1.84E−03 – 1.19E−02	4
Visual system			1.84E−03 – 1.99E−03	3

Of the 22 differentially expressed transcripts common to both exposure groups (Fig. 3B), the four most upregulated genes were genes encoding the human leukocyte antigens A and B (Supplementary Table 2: day 80 and day 140 testes, respectively: *HLA-A*: +1.75- and +1.67-fold change, *HLA-B*: +1.3- and +1.6-fold change). The two most downregulated genes in both groups (0–80T, 0–140T) were *POSTN* (periostin osteoblast specific factor) and ovin *ZFNLOC101104520* (zinc finger protein) (Supplementary Table 2: day 80 and day 140 testes, respectively: *POSTN*: −1.4- and −2.0-fold change, associated with tumour progression and *LOC101104520*: −1.3- and −1.2-fold change, respectively).

Confirmation of microarray changes was carried out by qPCR and comparable exposure-linked changes were observed for *POSTN* (decreased), *MHC class 1 HLA-B* (increased), and *PTGER3*(marginal decrease) (Supplementary Fig. 2 and Supplementary Table 2).

## Discussion

The current study identifies stages of male fetal gonad development in sheep that are developmentally sensitive to an EC mixture relevant to human exposure. In the current paradigm, exposure occurred when pregnant ewes grazed pastures fertilised with biosolids generated from processed human sewage sludge. Sertoli cell number in day 140 fetuses exposed throughout gestation (0–140 days) remained unchanged but Leydig cell CYP17A1 and CYP11A1 staining was reduced. In contrast, exposure for periods of 80 days limited to early, mid, or late gestation reduced both Sertoli cell numbers and Leydig cell CYP17A1 immuno-reactivity in day 140 male fetal testes, regardless of developmental stage. Of note is that the reduced proportion of interstitial cells positive for CYP17A1 in the 30–110 and 60–140T exposure groups is not reciprocated with CYP11A1. Since fetal testosterone concentrations were reduced only in the 60–140 exposure group, this likely reflects fetal Leydig cell activity and raises the possibility of an exposure-induced reduction in the proportion of CYP17A1-positive testosterone-secreting Leydig cells. Intriguingly, exposure limited to 30–110 days was not sufficient to reduce testosterone but still reduced the proportion of CYP17A1. The lack of an effect on CYP11A1 may reflect differential sensitivity of the steroidogenic enzymes and awaits further characterisation of these cells at this late stage of gestation.

Notably, exposure for the first 80 days of gestation resulted in a dramatic change in the day 140 testis transcriptome, whereas exposure for the last 80 days induced phenotypic differences manifest by male fetuses with reduced body mass, reduced AGD, smaller testes and adrenals, and reduced fetal testosterone. Indeed, these phenotypic observations are consistent with an anti-androgenic effect on the male fetus (Recabarren *et al.* 2008, Scully *et al.* 2018). Taken together, these data indicate that fetal testes transiently exposed to a cocktail of environmental contaminants either do not have time to adapt and/or that exposure alters the cellular composition of the testis between days 80 and 140. This raises the possibility that the transcriptomic changes observed may reflect some of the cellular changes in transient exposure groups.

### Transient vs continuous fetal testis exposure

Fetuses exposed to ECs for the first 80 days of gestation exhibited a greater change in testis transcriptome than those continuously exposed. Biological consistency can be drawn from similar transcriptomic changes in fetal ovaries examined from the same experimental paradigm. That is, a greater degree of ovarian transcriptomic change was observed in transient vs continuously exposed female fetuses (Lea *et al.* 2016). Furthermore, relative to animals exposed from mating to 110 days, exposure limited to the pre-conception period reduced the proportion of mid-gestation type 1a ovarian follicles, reduced small blood vessel numbers in the fetal thyroid and specific to the males, increased thyroid organ weights. (Bellingham *et al.* 2013, Hombach-Klonisch *et al.* 2013). It is plausible that in the current study, the lesser degree of testicular transcriptomic change reflects adaptation to a longer and continuous period of exposure with less severe consequences for the fetus. In contrast, altering the environment by moving pregnant ewes from biosolids to control pastures or vice versa requires the fetal testis to adapt at more advanced developmental stages with different longer-term consequences. This applies to all three transient exposure groups (0–80T, 30–110T, 60–140T), and the shorter adaptation period of the 0–80T group may account for the greater phenotypic changes observed at day 140. Although transcriptomics was not carried out on day 140 fetal testes transiently exposed during mid or late gestation, our observations on Sertoli and Leydig cell markers support this theory. This raises concerns that a transient environmental change during fetal development may adversely affect male reproduction during adulthood, a critically important area not yet explored within the current paradigm.

These data are consistent with three previous studies demonstrating developmental exposure effects on testes from mid-gestation male fetuses, neonatal day 1 males, and post-weaning ram lambs (Paul *et al.* 2005, Bellingham *et al.* 2012, Elcombe *et al.* 2021). Mid-gestation male fetuses continuously exposed to day 110 exhibited reduced Sertoli and Leydig cell numbers and this was reflected by reduced inhibin A and testosterone. In the current study, the lack of an effect at day 80 likely reflects the earlier developmental stage and/or the shorter period of continuous exposure. In neonatal lambs exposed *in utero* via the mother, and in adult males exposed via the mother and for a period of 7 months following parturition, testicular abnormalities were observed in a cohort of male offspring, for example fewer germ cells and Sertoli cell-only tubules (Bellingham *et al.* 2012, Elcombe *et al.* 2021).

### Anti-androgenic effects

Our observations of reduced fetal AGD and impaired testis development support studies linking these observations with chemical exposures during fetal developmental (Gray *et al.* 2006). Furthermore, a common masculinisation programming period has been identified in rats that occurs just after testis differentiation (Welsh *et al.* 2008). During this period, exposure to an androgenic chemical reduces AGD concomitantly with the induction of hypospadias and cryptorchidism. In developing male fetal sheep, prenatal androgen exposure mid-gestation also alters gonadal development although an increase in AGD has been reported (Manikkam *et al.* 2004, Padmanabhan *et al.* 2010). Notably, we have previously reported that female fetuses exposed to biosolids via the mother have an increased AGD and postulated that this may be androgenic (Lea *et al.* 2016, Dorman *et al.* 2018). In the current study, the reduction in AGD was specific to smaller male fetuses and this effect characterised only the late gestation exposure group. However, the fact that CYP17A1 and testosterone were significantly reduced and other organ weights (e.g. thyroid) were not affected in this group argues for a dysregulation of androgen production and target tissue response, probably exacerbated by the concomitant reduced growth. An increased risk of urogenital defects, like hypospadias, is associated with small for gestational age in humans and this is associated with altered placental androgen production (O’Shaughnessy *et al.* 2019). This raises the possibility that biosolids chemicals may disturb placental function/androgen production, leading to an increased risk of impaired masculinisation.

Importantly, during fetal life and after the proliferation of germ cells, the stages of development between the testis and ovary are fundamentally different. In the ovine (and human) ovary, two essential steps take place: meiosis initiation and blockage of the oocyte at the end of prophase I and follicle formation (Mandon-Pepin *et al.* 2003). In the testis, germ cells remain quiescent until puberty. Despite this observation, female fetuses had greater sensitivity to exposure during mid and late gestation rather than in early gestation as described for the males. This may reflect the earlier rate of development of the testis compared to the ovary (Smith *et al.* 2014, Wilhelm *et al.* 2007) and/or the differential sensitivity of the testis and ovary to specific chemical types. Of note is that maternal liver phthalate was increased specifically in the 0–80T group while specific changes in liver PCBs and PBDEs characterised the mid and late gestation groups (Lea *et al.* 2016).

### Transcriptomic analyses

Although direct causality cannot be established, the combined chemical measurements and transcriptomic studies suggest that the developing gonad is less well-equipped to respond to a transient change in environment, than to a consistent but abnormal change. In the current study, transcriptomic analyses highlighted drug metabolism as a key function of the differentially expressed genes in the 0–80T group but not the 0–140T group. Drug-metabolising enzymes such as the differentially expressed gonadal P450 (CYP) family genes reported in the current study are also reported to show altered expression during development (Hines 2008). We therefore propose that this may account for some of the differences between exposure groups. Also unique to this group were gene networks associated with development or disorders in a range of key body systems including haematological, connective tissue, neurological, cardiovascular, and metabolism. In contrast, high scoring networks in the 0–140T group were primarily linked to cell functions: signalling, interaction, assembly, and organisation, and these were also highlighted in the 0–80T group. Notably, our previous transcriptomic studies of biosolids-exposed fetal ovaries indicated that genes linked to drug metabolism were altered in the most perturbed 60–140T group (Lea *et al.* 2016). We postulated that activation of these genes following intermittent exposure of the fetus may impact later development and that this may account for the greater effect of transient exposures to biosolids. This raises the possibility that this may also apply to the developing male.

In the 0–80T group, the transcriptional regulator *SOHLH1,*associated with early spermatogonia differentiation and subsequent male fertility, was a top upregulated gene (Toyoda *et al.* 2014). In mice, *SOHLH1* is expressed in gonadal germ cells (fetal and post-natal) and is crucial for germ cell survival and spermatogenesis (Barrios *et al.* 2012). The dysregulation of *SOHLH1* may be associated with the altered androgen regulation of Sertoli cells and thus germ cell development. Furthermore, altered SOHLH1 may also be indirectly linked to exposure-induced changes in testicular drug metabolisms pathways. These areas constitute lines of further investigation.

The upregulation of HLA antigens in both exposure groups and the testicular cell-mediated/inflammatory gene networks identified may reflect exposure-induced changes in immune function (Bansal *et al.* 2018). Since immunological competence in fetal lambs develops progressively throughout gestation (Nalubamba *et al.* 2008), markers such as pattern recognition receptors (TLR10) and chemokine receptors (CXCR5) provide potential follow-up genes for further investigation. For both exposure groups, RXR function and PPAR∝ activation constituted the top toxicological network. The PPAR receptors, including PPAR∝ (NR1C1), are expressed in mammalian fetal testis (Froment *et al.* 2006) and the alpha form is implicated in Sertoli cell metabolism (Rouiller-Fabre *et al.* 2015). Since maternal liver phthalate levels were increased in the 0–80T group and rodent studies indicate phthalate toxicity effects on the testis are partly mediated by PPAR and RXR, phthalates may contribute to the effects of biosolids on the male ovine fetus (Ward *et al.* 1998, Ryu *et al.* 2008, Lea *et al.* 2016).

Androgen signalling was perturbed in the 0–80T group (DAVID) and this is consistent with the phenotypic effects observed in exposed male (reduced AGD, lowered testosterone, reduced fetal body, adrenal and testis mass) and female fetuses (e.g. increased AGD, increased testosterone, reduced fetal and uterus mass) (Lea *et al.* 2016). Angiogenic factors were also differentially affected across the two treated male groups along with classical cell signalling systems: retinoic acid, SMAD2/3, IGF1, ERK1/2/MAPK, and ErB1. The trend for downregulation of differentially expressed fetal testicular transcripts in the 0–80T group parallels the downregulation of fetal ovarian transcripts in fetuses from the same experiment (Lea *et al.* 2016). This may be reflective of an underlying epigenetic mechanism in both sexes. Interestingly, histone deacetylase signalling was altered in the 0–80T males, and in our previous study of the females, histone methylation genes were altered

## Conclusions

In conclusion, the data presented in this study demonstrate that the ovine fetal testis exhibits differential and temporal sensitivity to chemical mixtures present in pastures treated with biosolids fertiliser. We have demonstrated, for the first time, that a short period of environmental exposure applied for the first 80 days of gestation dramatically alters the fetal testis transcriptome at day 140. Furthermore, restricting the period of environmental change to the final 80 days of gestation induces phenotypic changes indicative of an anti-androgenic effect on the male fetus. Relative to the shorter periods of biosolids exposure, continuous exposure was associated with fewer differentially regulated genes and a lesser effect on the male phenotype (fetal, adrenal, testis mass, AGD, and fetal testosterone unaltered), we propose that a short period of exposure either does not allow the developing fetus to adapt and/or alters the cellular composition of the testis. Collectively, these data indicate that exposure of grazing ruminants to mixtures of chemicals contained in biosolids fertiliser may be linked to perturbations in testicular development. Given the relevance of the biosolids model to human exposures and the consumption of ruminant-derived food products, these data should be viewed as a concern for animal and human male reproductive health.

## Supplementary Material

Supplementary Figure S1: Immunolocalisation of anti-Mullerian hormone (AMH), proliferation marker Ki67 and the steroidogenic enzymes P450scc (CYP11A1) and P450c17 (CYP17A1). Positive staining is indicated by the brown colour (DAB), counterstained with hematoxylin (blue/purple). (A) AMH was localised to the Sertoli cells and Ki67 (B) localised primarily to the Sertoli cell nuclei. (C, D) The steroidogenic enzymes CYP11A1 (C) and CYP17A1 (D) were localised to the interstitial Leydig cell containing area of the testis. Inset images depict IgG controls. Scale bar = 50 ƲM.

Supplementary Figure S2: Validation of selected genes demonstrating treatment-related expression differences by microarray. Relative mRNA expression of Periostin osteoblast specific factor (POSTN), Prostaglandin E receptor 3 (PTGER3) and MHC Class 1 HLA-B in day 140 control (0-140C) and exposed (0-140T) fetal testis. (A) POSTN was signficantly lower in the 0-140T group (P=0.0004), (B) PTGER3 was unaltered between groups, (C) MHC Class 1 HLA-B was increased in the 0-140T group. qPCR data was analysed using the Roche LightCycler480 software and normalised by geNorm. Three housekeeping genes (GAPDH, HPRT, YWHAZ) were utilised testing for stability by geNorm, Normfinder and ANOVA analysis.

Supplementary Table 1: Quantitative PCR Probes and Primer sequences.

Supplementary Table 2

Supplementary Table 3

## Declaration of interest

The authors declare that there is no conflict of interest that could be perceived as prejudicing the impartiality of the research reported.

## Funding

This work was supported by the European Commission http://dx.doi.org/10.13039/501100000780 Framework 7 Programme (Contract No. 212885) and by the National Institutes of Health http://dx.doi.org/10.13039/100000002 (grant number R01 ES030374).

## Author contribution statement

R G L, C C, P A F, and K D S conceived of and designed the study. B F E and A B carried out the histological studies and testicular cell counts. C C, B M P, B L, E P, and L J designed and analysed the microarray results. L P, B M P, and R G L carried out all qPCR studies and analyses. Z Z carried out all chemical analyses and academic input in interpreting chemical effects. R G L and K D S wrote the paper. P A F co-ordinated the REEF EU FP7 project which funded the work and R G L, K D S, and C C were work-package leaders.
