# Supplementary material for: Ovine fetal testis stage-specific sensitivity to environmental chemical mixtures
Source: Reproduction. 2022 Jan 11;163(2):119–31. doi: 10.1530/REP-21-0235 (PMC8859917; doi:10.1530/REP-21-0235)
Supplement: Supplementary Figure S1: Immunolocalisation of anti-Mullerian hormone (AMH), proliferation marker Ki67 and the steroidogenic enzymes P450scc (CYP11A1) and P450c17 (CYP17A1). Positive staining is indicated by the brown colour (DAB), counterstained with hematoxylin (blue/purple). (A) AMH was localised [file supplementary_figure_1.pdf]

Supplementary Figure S1:

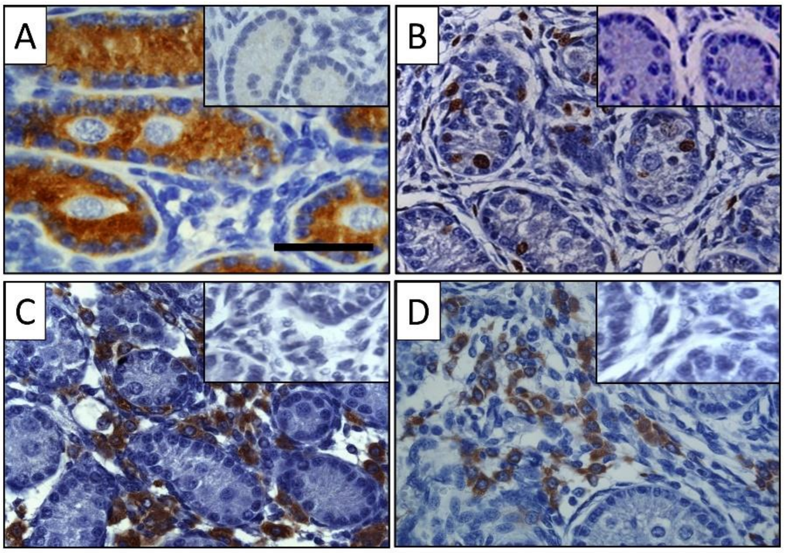

Supplementary Figure S1: Immunolocalisation of anti-Mullerian hormone (AMH), proliferation marker Ki67 and the steroidogenic enzymes P450scc (CYP11A1) and P450c17 (CYP17A1). Positive staining is indicated by the brown colour (DAB), counterstained with hematoxylin (blue/purple). (A) AMH was localised to the Sertoli cells and Ki67 (B) localised primarily to the Sertoli cell nuclei. (C, D) The steroidogenic enzymes CYP11A1 (C) and CYP17A1 (D) were localised to the interstitial Leydig cell containing area of the testis. Inset images depict IgG controls. Scale bar = 50 υM.
