# Supplementary material for: Ovine fetal testis stage-specific sensitivity to environmental chemical mixtures
Source: Reproduction. 2022 Jan 11;163(2):119–31. doi: 10.1530/REP-21-0235 (PMC8859917; doi:10.1530/REP-21-0235)
Supplement: Supplementary Figure S2: Validation of selected genes demonstrating treatment-related expression differences by microarray. Relative mRNA expression of Periostin osteoblast specific factor (POSTN), Prostaglandin E receptor 3 (PTGER3) and MHC Class 1 HLA-B in day 140 control (0-140C) and exposed (0-1 [file supplementary_figure_2.pdf]

Supplementary Figure S2:

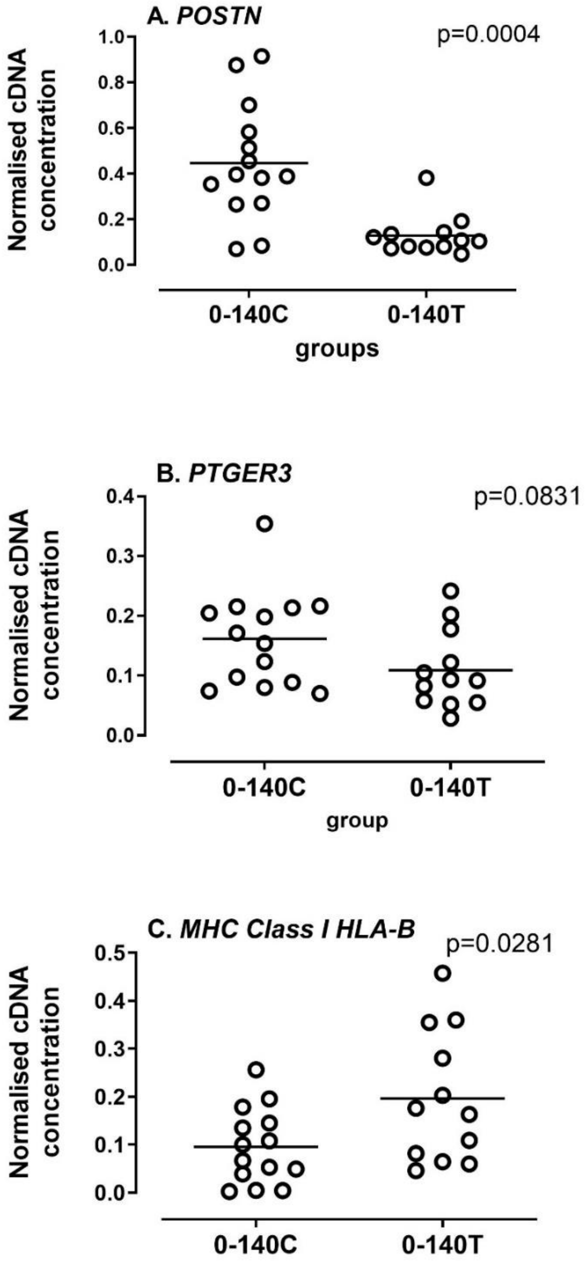

Supplementary Figure S2: Validation of selected genes demonstrating treatment-related expression differences by microarray. Relative mRNA expression of Periostin osteoblast specific factor (*POSTN*), Prostaglandin E receptor 3 (*PTGER3*) and *MHC Class 1 HLA-B* in day 140 control (0-140C) and exposed (0-140T) fetal testis. (A) *POSTN* was signficantly lower in the 0-140T group (P=0.0004), (B) *PTGER3* was unaltered between groups, (C) *MHC Class 1 HLA-B* was increased in the 0-140T group. qPCR data was analysed using the Roche LightCycler480 software and normalised by geNorm. Three housekeeping genes (*GAPDH, HPRT, YWHAZ*) were utilised testing for stability by geNorm, Normfinder and ANOVA analysis.
