# Supplementary material for: Ovine fetal testis stage-specific sensitivity to environmental chemical mixtures
Source: Reproduction. 2022 Jan 11;163(2):119–31. doi: 10.1530/REP-21-0235 (PMC8859917; doi:10.1530/REP-21-0235)
Supplement: Supplementary Table 1: Quantitative PCR Probes and Primer sequences. [file supplementary_table_1.pdf]

Supplementary Table 1: Quantitative PCR Probes and Primer sequences.

Target	Primer sequence	GenBank accession number
GAPDH	F: GGTTCACGCCCATCACA	NM_001190390
	R: ACTACCATGGAGAAGGCTGG	
	PR: AGAGGGTCATCATCTCTGCACCTTCT	
HPRT	F: GAACGGCTGGCTCGAG	EE751310
	R: CCAACAGGTCGGCAAAG	
	PR: AATGTGATGGCCACCCATCTCCT	
YWHAZ	F: GGAGCCCGTAGGTCATCTTG	AY970970
	R: CTCGAGCCATCTGCTGTTTTT	
	PR: CAGCACCTTCCGTCTTTTGCTCAATACTGGAGA	
POSTN	F: AATGCATGCAAGGAGTTACC	XM_004012108.4
	R: TGCCAGTAAACCCACATTTT	
	PR: CCCATTTTTATTTACCCATTTCCCA	
PTGER3	F: AATCACACGTCAGTTGAGCA	AF035417
	R: CCAGGCGAACAGCTATTAAG	
	PR: CACAGAAAATCAGGATGAGTGCAAC	
MHC Class I HLA-B	F: AGGAGACGCAGGGAACTAAGG	AJ874684
	R: GTAGCCGCGCAGGTTGTT	
	PR: CACTGCACTGACTTTCCGAGCGAACCT	
